# Proteinoid Microspheres as Protoneural Networks

**DOI:** 10.1021/acsomega.3c05670

**Published:** 2023-09-12

**Authors:** Panagiotis Mougkogiannis, Andrew Adamatzky

**Affiliations:** Unconventional Computing Laboratory, UWE, Bristol BS16 1QY, U.K.

## Abstract

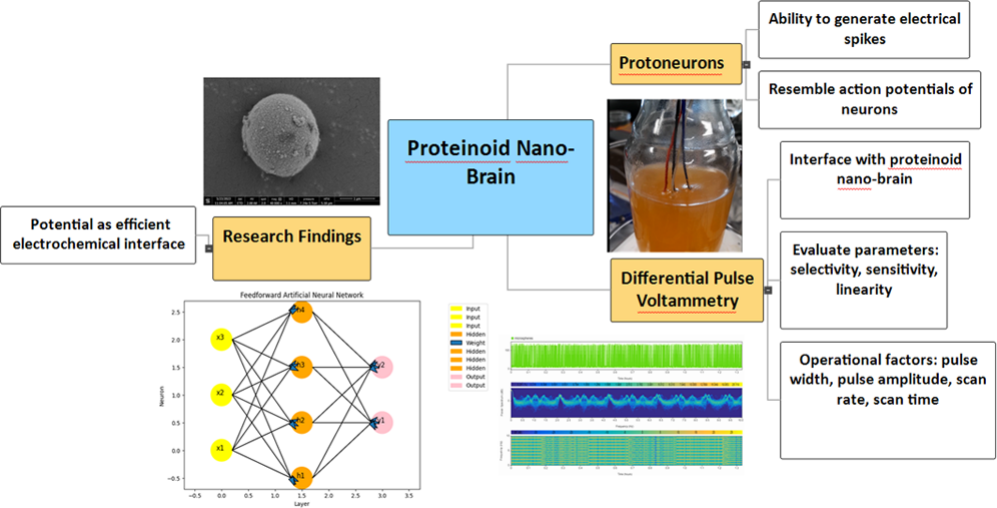

Proteinoids, also known as thermal proteins, possess a fascinating ability to generate microspheres that exhibit electrical spikes resembling the action potentials of neurons. These spiking microspheres, referred to as protoneurons, hold the potential to assemble into proto-nanobrains. In our study, we investigate the feasibility of utilizing a promising electrochemical technique called differential pulse voltammetry (DPV) to interface with proteinoid nanobrains. We evaluate DPV’s suitability by examining critical parameters such as selectivity, sensitivity, and linearity of the electrochemical responses. The research systematically explores the influence of various operational factors, including pulse width, pulse amplitude, scan rate, and scan time. Encouragingly, our findings indicate that DPV exhibits significant potential as an efficient electrochemical interface for proteinoid nanobrains. This technology opens up new avenues for developing artificial neural networks with broad applications across diverse fields of research.

## 1 Introduction

While there are numerous prototypes of organic electronic devices,^1−4^ very few, if any, demonstrate substantial degrees of stability or biocompatibility.^5^ This is why we propose to explore thermal proteinoids,^6^ a unique class of organic chemical compounds, as a potential substrate and architecture for future non-silicon massive parallel computers. Proteinoids, also known as thermal proteins, are derived by subjecting amino acids to high temperatures until they reach their melting point, leading to polycondensation and the formation of polymeric chains.^6^ This polymerization process occurs in the absence of solvents, initiators, or catalysts under an inert atmosphere, typically at temperatures ranging from 160 to 200 °C. Specifically, the trifunctional amino acids, such as glutamic acid, aspartic acid, or lysine, undergo cyclization at elevated temperatures and serve as solvents and initiators for the polymerization of other amino acids.^6,7^ The intriguing capacity of proteinoid microspheres to generate action-potential-like spikes and spike trains has led to their consideration as analogues of protoneurons, which are neuron-like cells that function without metabolic processes.^8−10^ Proteinoids do not possess metabolic machinery, but the observed spiking activity is fueled by transmembrane proton flux, which is made possible by the membrane structure of proteinoid microspheres. The proteinoid vessels consist of interior aqueous pools that are acidic, and these pools are encapsulated by permeable peptide membranes. The pH gradient mentioned can facilitate the movement of H^+^ ions through concentration and electrical potential differences, thereby generating the necessary energy for electrochemical signaling.

We explore the concept of nanobrains (PNBs)^11,12^ to evaluate the feasibility of proteinoid microspheres for the physical imitation of artificial neuron networks (ANNs).^13^ Namely, we aim to imitate neuronal responses to external stimuli^14−18^ in PNBs. We use differential pulse voltammetry (DPV) to assess the capabilities of PBNs for pattern recognition.

In this study, we employ techniques from the fields of electrochemical neuroscience, artificial neural networks (ANNs), and pattern recognition to analyze the spikes generated by PNBs. Specifically, we utilize differential pulse voltammetry (DPV) to measure the electrical signals produced by PNBs. Through this analysis, we aim to understand the behavior of PNBs and evaluate their capability for pattern recognition. Our objective is to review the existing literature that explores the relationship between PNBs and ANNs, identify any gaps or limitations in the current understanding, and propose a research methodology to investigate the potential of PNBs in the field of pattern recognition.^19−21^

Now, let us delve into the examination of the neural networking capabilities of biological neurons. A group of researchers at NIST (National Institute of Standards and Technology) has made significant advancements in this area by developing an artificial neuron that exhibits an astonishing firing rate of 100 billion times per second.^13^ This remarkable speed surpasses the firing rate of a human brain cell by approximately tenfold. The research article highlights the use of niobium nitride, a superconducting material, in the artificial neuron. This material allows the neuron to switch between two distinct electrical resistance states when exposed to magnetic fields. The article discusses the possibilities and challenges associated with creating “neuromorphic” hardware that emulates the complex functioning of the human brain.^13^

In their research, Wan et al. presented a breakthrough in the field of artificial neurons by showcasing the functionality of an artificial sensory neuron capable of gathering optic and pressure data from photodetectors and pressure sensors, respectively.^19^ This neuron can transmit the combined information through an ionic cable and integrate it into postsynaptic currents using a synaptic transistor. The study highlights the significance of synchronizing two sensory cues, as it activates the artificial sensory neuron at different levels, enabling the control of skeletal myotubes and a robotic hand. Furthermore, the research demonstrates that the artificial sensory neuron enhances recognition capabilities for combined visual and haptic cues through the simulation of a multitransparency pattern recognition task.^19^

In their study, Boddy et al.^120^ employ artificial neural networks (ANNs) to effectively identify and classify marine phytoplankton using flow cytometry data, showcasing the capability of ANNs in recognizing patterns in biological data.^20^ The article provides an overview of the structure and training process of three types of ANNs: backpropagation (multilayer perceptron), radial basis function (RBF), and learning vector quantization. These ANNs utilize supervised learning techniques and are well-suited for biological identification purposes. Additionally, the study highlights the effectiveness of Kohonen self-organizing maps (SOM) and adaptive resonance theory (ART) as classification methods.^20^

In their research, Syed and colleagues introduce a groundbreaking concept that goes beyond the traditional fixed feedforward operation commonly found in contemporary artificial neural networks.^22^ The study presents a novel class of synthetic neurons capable of adapting their functionality in response to feedback signals from neighboring neurons. These synthetic neurons demonstrate the ability to emulate complex brain functions, including spike frequency adaptation, spike-timing-dependent plasticity, short-term memory, and chaotic dynamics.^22^

Baluska et al. explore the evolutionary perspective of biomolecular structures and processes that contribute to the emergence and maintenance of cellular consciousness.^11^ The proposition suggests that subcellular components, such as actin cytoskeletons and membranes, play a crucial role in nanointentionality. This is attributed to the inherent structural adaptability of individual biomolecules, extending beyond cellular boundaries.^11^

The present paper focuses on exploring the capabilities of proteinoid nanobrains (PNBs) in processing signals obtained from a differential pulse voltammetry (DPV) electrode and their potential for pattern recognition, drawing inspiration from artificial neural networks (ANNs).^22−24^ The objective of this study is to investigate the ability of PNBs to detect spikes induced by DPV signals. We aim to assess the responsiveness of PNBs to DPV signals and their capacity to generate ANNs for pattern recognition purposes. Experimental results are presented, evaluating the pattern recognition performance of PNBs using DPV signals. The paper concludes by discussing the implications of the findings and providing recommendations for future research.

## 2 Methods

**Figure 1:**
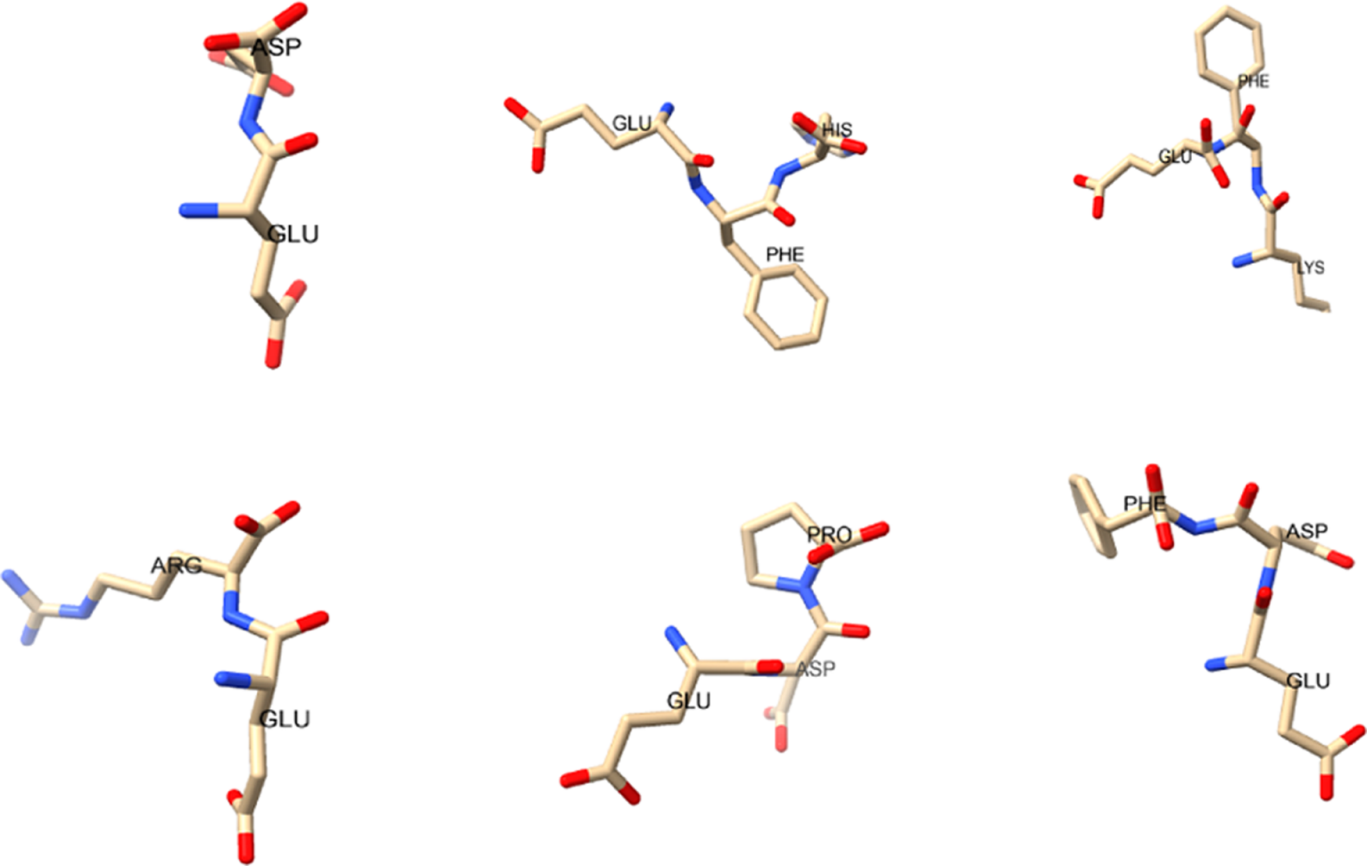
Chemical structures of L-Glu:L-Asp, L-Glu:L-Phe:L-His, L-Lys:L-Phe:L-Glu, L-Arg,L-Glu, L-Glu:L-Asp-L-Pro, and L-Glu:L-Asp:L-Phe.

High-purity amino acids, including l-phenylalanine, l-aspartic acid, l-histidine, l-glutamic acid, and l-lysine (Figure 1), were acquired from Sigma Aldrich with a purity exceeding 98%. The synthesis of proteinoids followed previously established methods.^25^ The structural analysis of the proteinoids was conducted using scanning electron microscopy (SEM) with FEI Quanta 650 equipment. Characterization of the proteinoids was performed using Fourier transform infrared (FT-IR) spectroscopy.^25^

To measure the electrical activity of the proteinoids, iridium-coated stainless steel subdermal needle electrodes (Spes Medica S.r.l., Italy) and a high-resolution data logger equipped with a 24-bit A/D converter (ADC-24, Pico Technology, U.K.) were used. The electrodes were configured in pairs to measure the potential difference between them, with an interelectrode distance of approximately 10 mm. Electrical activity was recorded at a sampling rate of one sample per second. The data logger recorded multiple measurements (typically up to 600 per second) and stored the mean value for analysis.

Differential pulse voltammetry (DPV) is used to monitor dynamic electrical activity. In DPV, the potentiostat applies voltage pulses with fixed amplitude increments and includes resting periods for integration. The current is sampled immediately before and after each pulse. The difference between these two samples provides the Faradaic current resulting from the redox reactions induced by the pulse. When the potential is varied across a certain range, the voltammogram shows transient oxidation or reduction current peaks, which indicate spikes in electrical activity. The proteinoid microspheres display distinct current peaks as a result of redox reactions occurring within chemical species. The nature of the electrical events occurring within the dynamic proteinoid vesicle networks can be determined by analyzing the timing, amplitude, and shape of the observed current spikes. DPV’s high sensitivity enables the detection of emerging spike behaviors in self-assembled biomolecular systems.

Differential pulse voltammetry (DPV) can be used to take accurate measurements with the Zimmer & Peacock Anapot EIS. The Anapot EIS provides users with the flexibility to define measurement parameters for conducting differential pulse voltammetry (DPV) experiments. In order to perform a DPV measurement, several key parameters need to be specified, as follows: the equilibrium time is set to 100 s, the potential scan starts at −8 V, the potential scan ends at 8 V, the potential step size is set to 0.001 V, the pulse amplitude is set to 0.2 V, the pulse width is specified as 0.08 s, and the scan rate is set to 0.001 V/s. During the measurement process, the Anapot EIS applies brief pulses to the working electrode in small steps. It measures the current response twice in each step, capturing the current values before and after the pulse. This process is repeated until every phase of the potential scan is completed.

By precisely controlling the measurement parameters and acquiring current response data at different potentials, the Anapot EIS enables comprehensive analysis and characterization of samples through differential pulse voltammetry.

## 3 Results

**Figure 2:**
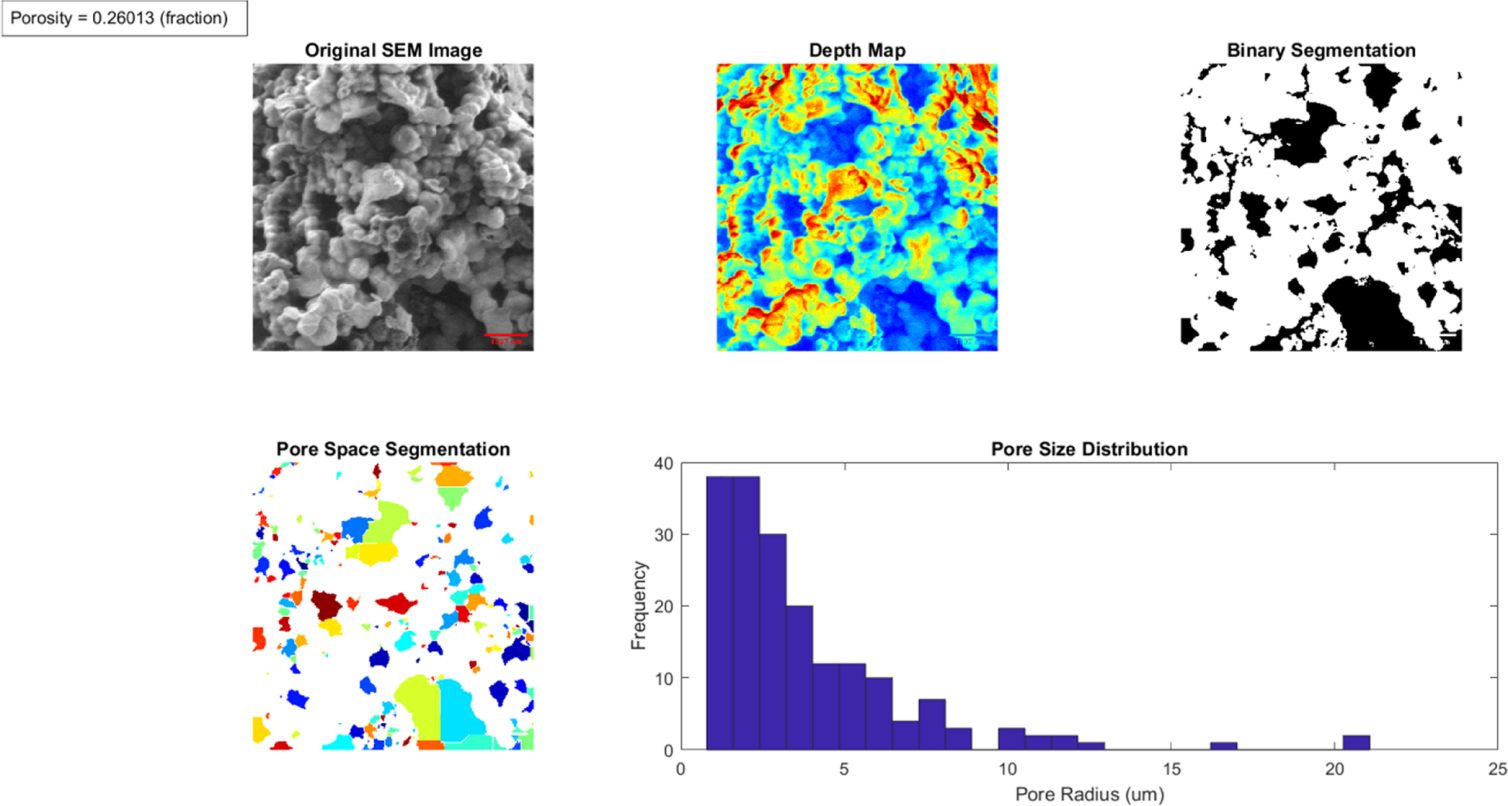
Porosity of proteinoids. This graph shows the average porosity (3.7982 μm) of proteinoids, represented by a depth map, binary segmentation, pore space segmentation, and pore size distribution in μm.^26^

Scanning electron microscopy revealed the complex proteinoid molecular network tuned to 1337 nm porosity (Figure 2). The network of molecules observed in our experiments appears to show some morphological similarity to neural cultures.^27^

**Figure 3:**
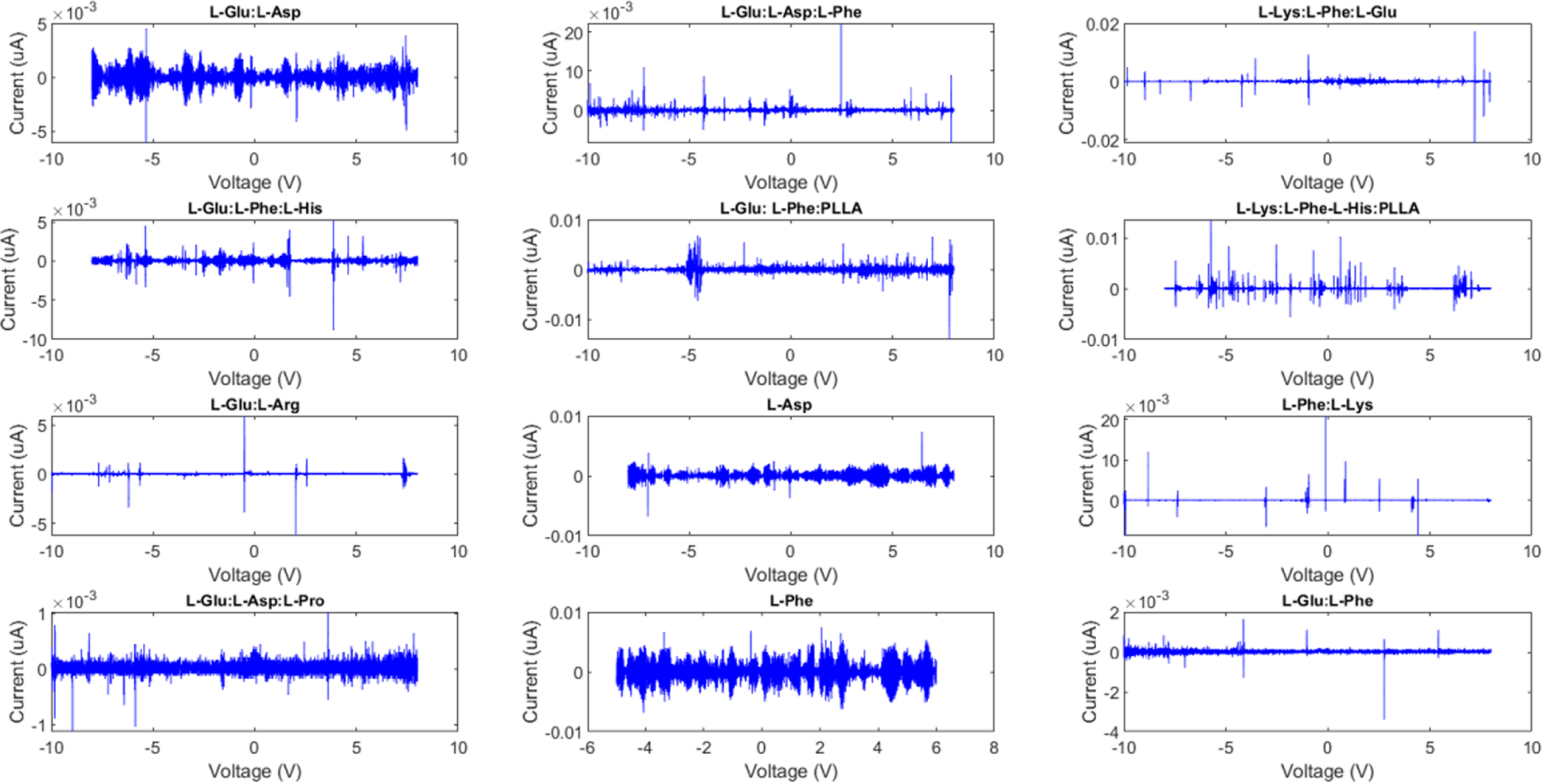
DPV measurements of 12 different proteinoids. The height of each peak is proportional to the number of microspheres present in the sample.

The results of Figure 3 suggest that proteinoids behave as electrical semiconductors, likely due to their amino acid chain structure. Although the current oscillations displayed in Figure 3 are fascinating in terms of their dynamics, it is necessary to gather more substantial evidence in order to fully understand the electrical properties and conduction mechanisms in proteinoids. The suggestion of semiconducting behavior in this case, which is solely based on temporal current fluctuations, is still speculative without further electrical, structural, and theoretical analyses. This type of electrical activity is reminiscent of that observed in neurons within the nervous system.^28−31^ Although the level of electrical activity is lower than that of neurons, the similarity in its behavior is intriguing. Despite this, the results of Figure 3 suggest that the electrical nature of proteinoids may exhibit properties similar to what is observed in biological cells.^21,32,33^ The data from Figure 3 is further supported by our previous research that proteinoids could synchronize electrical activity.^21,25^

**Figure 4:**
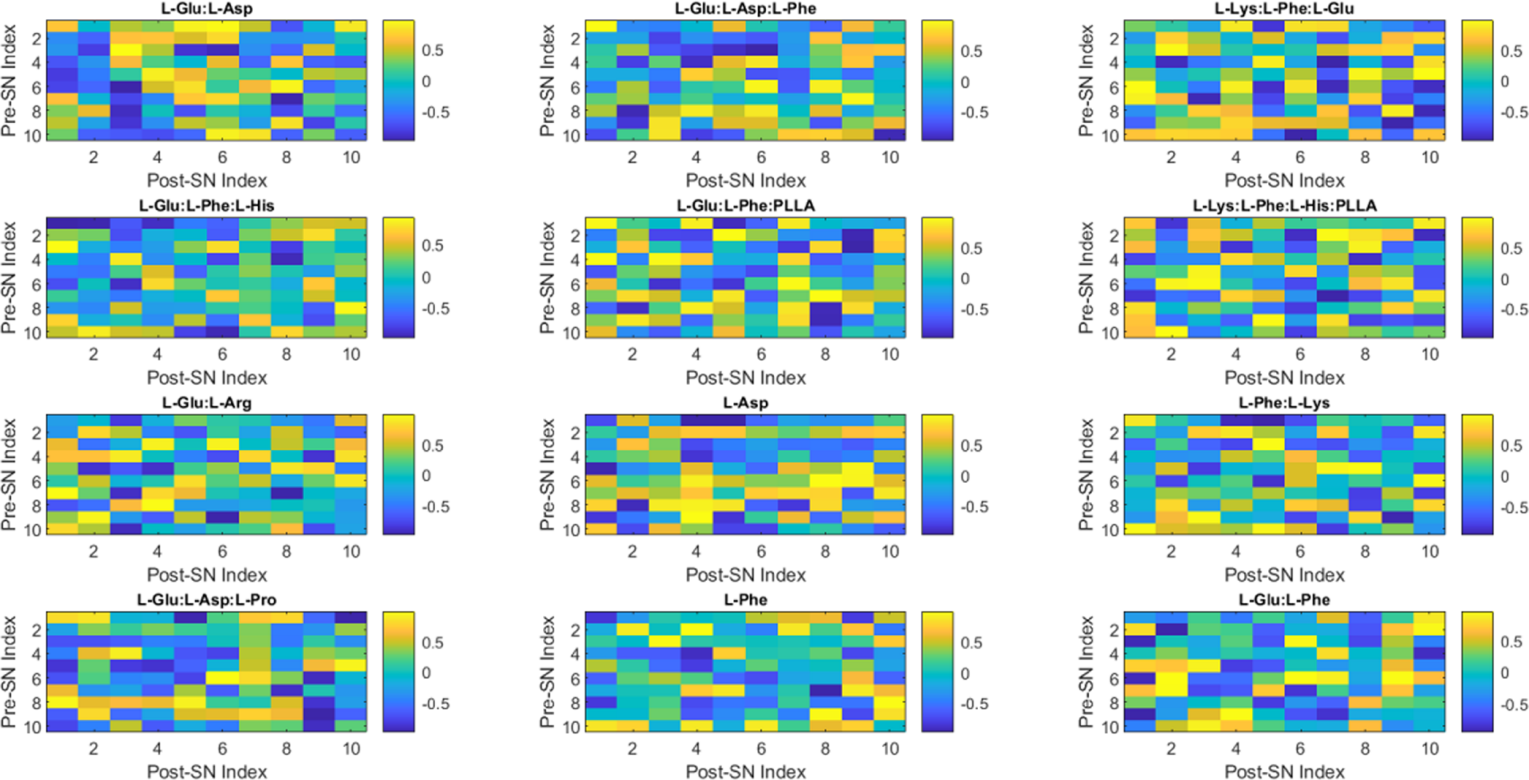
Presented color map depicts the PSI and PPI values of various proteinoids. PSI, or postsynaptic index, quantifies the chemical or functional potency of interneuronal connections within a network. PPI stands for post-postsynaptic index. It quantifies the efficacy of interneuronal connections in a given network. Darker colors of blue indicate elevated PSI values, whereas lighter colors of green indicate elevated PPI values. The map illustrates the correlation between postsynaptic and presynaptic neurons and their influence on proteinoid function.

Figure 4 depicts the relationship between presynaptic and postsynaptic neurons and their impact on proteinoid activity. The terms presynaptic and postsynaptic refer to the two sides of a synapse, which is the point of connection between two neurons or between a neuron and a target cell. The presynaptic neuron releases neurotransmitters, which are chemical messengers facilitating intercellular communication. The postsynaptic neuron receives and responds to the neurotransmitter by either firing or not firing an action potential.^34^ Although proteinoid microspheres are capable of exhibiting spike-like electrical activity, further investigation is necessary to determine whether similar synaptic communication occurs among proteinoid vesicles. The labeling of the postsynaptic and presynaptic neurons in Figure 4 serves as an initial abstract model for potential interproteinoid signaling. The PSI and PPI metrics are computational representations of proteinoid electrical behaviors within neural simulation frameworks. However, it is important to note that they do not provide confirmation of actual synaptic-like functions. To establish a connection between the emergent activities of interconnected proteinoid vesicles and neural systems, it is necessary to conduct a more comprehensive analysis of the chemical and electrical interactions between them, as well as their temporal dynamics.

The computational model shown in Figure 4 is an artificial neural network designed to replicate the observed signaling behaviors in networks of interconnected proteinoid microspheres. The input data comprises electrical potential measurements taken from proteinoid samples. These samples exhibit spikes that bear resemblance to neuronal action potentials. The connectivity between individual microspheres is modeled using artificial neural network (ANN) nodes, which represent virtual proteinoid vesicles. The weighted connections between these nodes indicate the strength of coupling between the vesicles. Nevertheless, the investigation into the physical mechanisms underlying the self-assembly and signal propagation of proteinoid networks is still ongoing. The synaptic weights in the model represent an abstract representation of the experimental intervesicular communication. This communication likely involves diffusive molecular signals or electrical field effects. The artificial neural network (ANN) utilizes temporal coding to transform the voltage spikes of the proteinoid into distinct spike events for every virtual node. The rules of unsupervised learning involve adjusting the simulated synaptic weights in order to capture the co-activation patterns between proteinoid signals. The self-organized topology that emerges provides valuable insights into the collective behaviors exhibited by the proteinoid microsphere networks observed in experiments.

**Figure 5:**
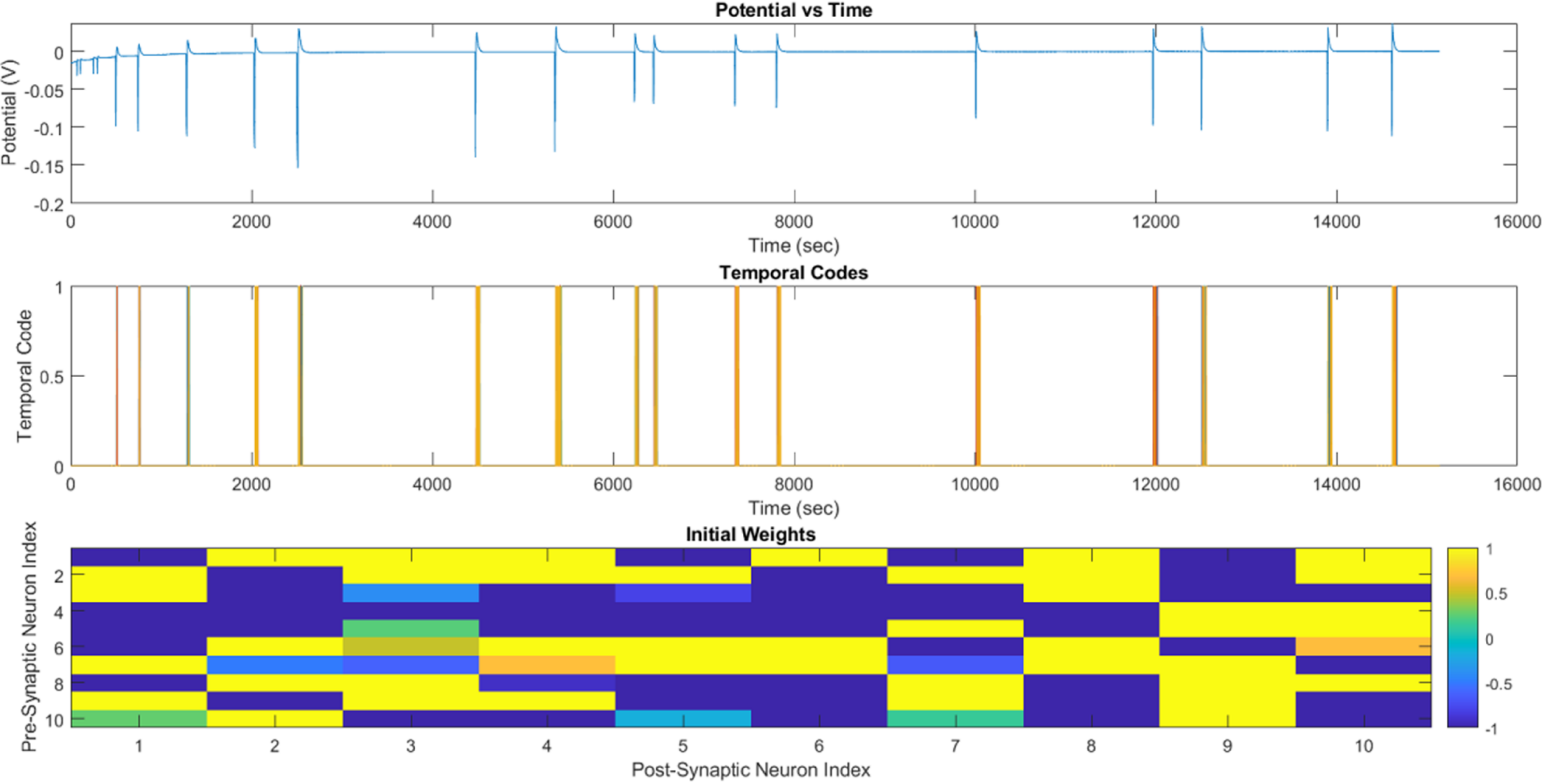
Potential of the proteinoid L-Glu:L-Phe when electrically stimulated reveals a temporal code that can be seen in the plots of initial weight and pre- and postsynaptic indices over time.

Initially, the synaptic weights of a 10-neuron network were randomly initialized within the range of −1 to 1, as presented in Table 1. Furthermore, the way in which the input is converted into an output by the activation function (as depicted in Figure 5) may be understood as a depiction of the information exchange among neurons in the nervous system, wherein a greater input would yield a correspondingly higher output. Furthermore, the temporal codes may be regarded as a depiction of the action potential within the nervous system, in which the potential must attain a threshold prior to the enhancement of temporal codes and the consequent activation of the output. Based on the data presented in Table 2, it can be observed that the proteinoids exhibited a considerable range in the quantity of spikes they generated. The presence of this phenomenon was demonstrated through the fluctuating quantity of spikes that were detected in the voltage–current graphs. The findings indicate that the mean number of spikes observed was 385.8, with a range spanning from 8 spikes for the L-Glu:L-Asp:L-Pro sample to 900 spikes for the L-Phe sample. The time duration metrics of the previously mentioned spikes varied from 20.71 s for L-Phe to 2541 s for L-Glu:L-Asp:L-Pro. The data indicates that the proteinoids exhibited a mean interspike interval of 425.30 s.

**Table 1:** Initial Values of the W Matrix for a Temporal Coding Neural Network with 10 Neurons and Random Weights

–1.0	1.0	1.0	1.0	–1.0	1.0	–1.0	1.0	–1.0	1.0
1.0	–1.0	1.0	1.0	1.0	–1.0	1.0	1.0	–1.0	1.0
1.0	–1.0	–0.4	–1.0	–0.8	–1.0	–1.0	1.0	–1.0	–1.0
–1.0	–1.0	–1.0	–1.0	–1.0	–1.0	–1.0	–1.0	1.0	1.0
–1.0	–1.0	0.2	–1.0	–1.0	–1.0	1.0	–1.0	1.0	1.0
–1.0	1.0	0.5	1.0	1.0	1.0	–1.0	1.0	–1.0	0.7
1.0	–0.5	–0.6	0.7	1.0	1.0	–0.7	1.0	1.0	–1.0
–1.0	1.0	1.0	–0.9	–1.0	–1.0	1.0	–1.0	1.0	1.0
1.0	–1.0	1.0	1.0	–1.0	–1.0	1.0	–1.0	1.0	–1.0
0.3	1.0	–1.0	–1.0	–0.2	–1.0	0.1	–1.0	1.0	–1.0

**Table 2:** Proteinoid Spike Characteristics: Number of Spikes, Mean Interspike Intervals (s), and Frequency of SSpiking (mHz); the Series of Measurements Obtained for These Proteinoids Showed a Threshold of Spiking at 0.0005 μA with a Minimum Peak Distance of 5 s

proteinoid	number	mean	frequency
	of	interspike	of
	spikes	interval (s)	spiking (mHz)
L-Glu:L-Asp	726	22.24	44.97
L-Glu:L-Asp:L-Phe	359	50.48	19.80
L-Lys:L-Phe:L-Glu	210	85.75	11.66
L-Glu:L-Phe:L-His	382	42.21	23.69
L-Glu:L-Phe:PLLA	555	32.71	30.57
L-Lys:L-Phe:L-His:PLLA	195	77.29	12.94
L-Glu:L-Arg	29	544.68	48.28
L-Asp	779	20.71	36.26
L-Phe:L-Lys	28	666.11	1.50
L-Glu:L-Asp:L-Pro	8	2541.00	0.39
L-Phe	900	12.32	81.15
L-Glu:L-Phe	12	1412.55	0.71

The combination of L-Glu, L-Asp, and L-Pro in a ratio of L-Glu:L-Asp:L-Pro resulted in a lower number of spikes, 8 compared to other combinations. Additionally, the mean interspike interval (2541.00 s) for these three combinations was slightly higher than that of the other combinations. Proteinoids can be used to make interpretations and analogies of the nervous system based on this mathematical relationship.

Let  be a vector of time values in seconds,  be a matrix of potential values in volts for 12 samples,  be the number of neurons in the ANN,  be the time window for temporal coding in seconds,  be the threshold for spike detection in volts, *c* ∈ {0, 1}^*N*×*n*^ be a matrix of temporal codes for each neuron over time, and *W* ∈ [−1, 1]^*N*×*N*^ be a matrix of synaptic weights between neurons. Then, for each sample *i* = 1, ···, 12, we have *c*_*j,i*_ = 1_[*p*_*j,i*_>θ]_(*j*)·(*T* − min_*k*∈[1,*n*]_{*p*_*k,i*_:*p*_*k,i*_ > θ}) where 1_[*p*_*j*,*i*_>θ]_(*j*) is an indicator function that returns 1 if *p*_*j*,*i*_ is greater than θ, and 0 otherwise.

Neurons are distinguished by their unique temporal coding, input parameters, and synaptic weights. When the temporal code (*c*_:,*j*_) exceeds the threshold parameter (θ), the neuron representation fires an action potential through its axonal connections, similar to a real neuron. The proteinoid neurons bear a resemblance to the neurons in an actual nervous system. The synaptic weights (*W*) in a neural network are similar to the synapses in a biological nervous system, as they govern the potency of the link between axons and dendrites.

Figures 3 and 5 differ in the stimulation level. Figure 3 utilizes DPV, whereas Figure 5 employs a power source that supplies a stable voltage through the proteinoid solution. According to the findings of the current research, proteinoids are capable of interpreting and responding to various forms of stimulation. When stimulated with DPV (Figure 3), the proteinoid solution unexpectedly produced oscillating signals as if it were a nervous system analogue. Again, unexpectedly, when the proteinoid solution was stimulated with a stable power source (Figure 5), discrete signals were produced. Similar to a nervous system, the proteinoid solution was able to interpret and respond to various forms of stimulation, as indicated by the results. In addition, it appears that the stability of the power source may affect the modulation of the response. This phenomenon can be attributed to the proteinoids’ ability to distinguish between the DPV and the stabilized power source and to react accordingly.

**Figure 6:**
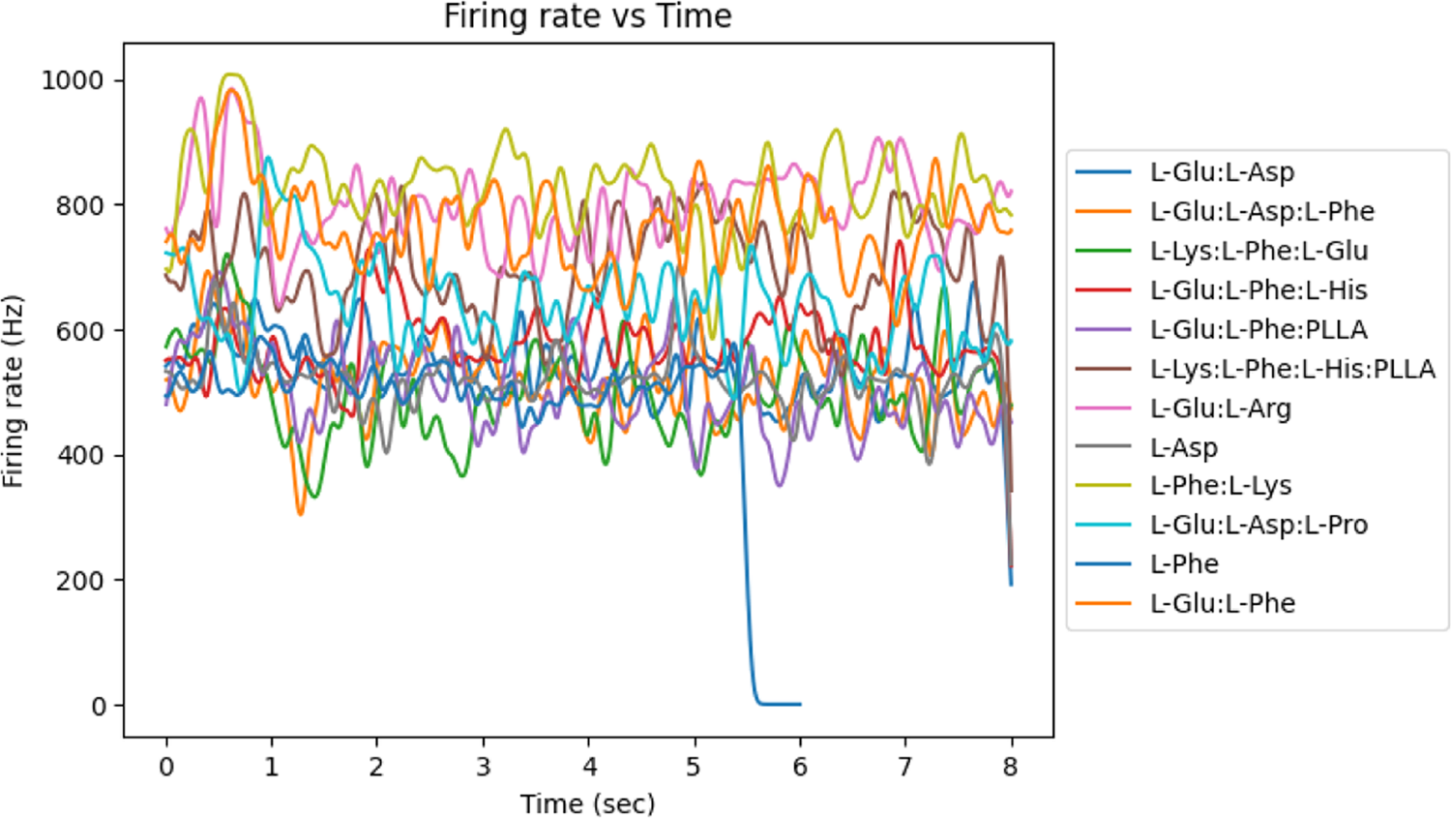
L-Phe:L-Lys exhibited the highest firing rate at 1008.2875 Hz. L-Phenylalanine exhibits the lowest firing rate at 591.6153 Hz. The firing rates (in Hz) for the proteinoids “L-Glu:L-Asp”, “L-Glu:L-Asp:L-Phe”, “L-Lys:L-Phe:L-Glu”, “L-Glu:L-Phe:L-His”, “L-Glu:L-Phe:PLLA”, “L-Lys:L-Phe:L-His:PLLA”, “L-Glu:L-Arg”, “L-Asp”, “L-Phe:L-Lys”, “L-Glu:L-Asp:L-Pro”, “L-Phe”, and “L-Glu:L-Phe” are 535.4877, 436.2721, 542.9443, 567.0562, 498.2888, 650.4798, 732.9516, 529.072, 768.2345, 617.3223, 491.5065, and 665.2995, respectively.

The results of this study indicate that proteinoid microspheres demonstrate an association between molecular properties and firing rates, as presented in Figure 6. The firing rate increases significantly with increases in molecular weight and peptide length. This correlation between structural parameters and electrical activity alludes to the possibility of proteinoid microspheres acting as analogues of neurons and forming the basis of a primitive nervous system. The firing rate of proteinoid microspheres can be used as an indicator of their ability to replicate the functions of a neuron, such as transmitting information. This provides evidence for the potential use of proteinoid microspheres as substrates for artificial neural networks.

The strength of the linear model suggests that further research should focus on a deeper understanding of the underlying mechanism that leads to the correlation between the molecular parameters and firing rate. Moreover, the linear model could be used to predict the firing rates of proteinoid microspheres for improved design of artificial neural networks.

**Figure 7:**
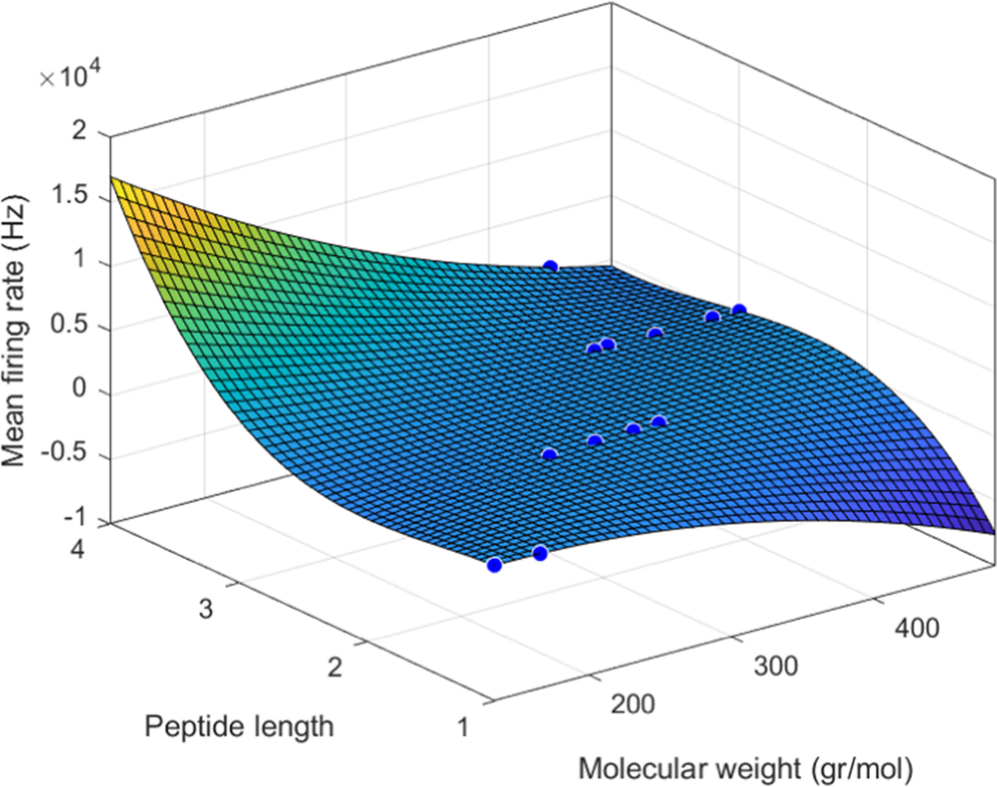
For 12 distinct proteinoid microspheres, a QSAR model was used to predict the mean firing rates in Hz, peptide length, and molecular weight in g/mol.

The linear model that best fits our data is represented by the following equation. Figure 7 shows the scatter plot of the firing rate versus the molecular weight and the peptide length, along with the regression plane of the model
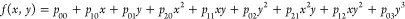
Coefficients (with 95% confidence bounds)
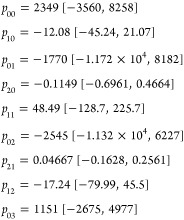


The present research indicates that proteinoid microspheres exhibiting higher mean firing rates, predicted QSAR, and % deviations are more effective in transmitting signals than those with lower values. The observed phenomenon can be attributed to the increased capacity of the larger microspheres to accommodate a higher quantity of proteinoids, leading to a greater number of active neurons. Higher QSAR values suggest that microspheres are more likely to initiate a neuronal cascade, which is crucial for effective signal transmission. The mean firing rate is a crucial parameter for assessing the efficacy of a neuron in signal transmission, representing the average number of firings within a specified time frame. QSAR prediction refers to the anticipated capacity of a neuron to activate, derived from experimental data. The % deviation represents the disparity between the anticipated outcome and the observed outcome of the experiment. Proteinoid microspheres have potential as a substrate for developing artificial brains and unconventional computing devices due to their ability to generate and transmit electrical activity and react to external stimuli, as reported by certain sources.^35^ They can form programmable networks through pores and tubes. This study of proteinoid oscillations provides insights into their molecular dynamics and intermolecular interactions (Table 3).

**Table 3:** Mean Firing Rate and Predicted QSAR of Different Proteinoid Microsphere Samples

sample	mean firing rate (Hz)	predicted QSAR (Hz)
L-Glu:L-Asp	535.4877	536.0542
L-Glu:L-Asp:L-Phe	436.2721	492.8753
L-Lys:L-Phe:L-Glu	542.9443	563.6253
L-Glu:L-Phe:L-His	567.0562	521.7084
L-Glu:L-Phe:PLLA	498.2888	551.9483
L-Lys:L-Phe:L-His:PLLA	650.4798	–901.3635
L-Glu:L-Arg	732.9516	–2041.8
L-Asp	529.072	723.4966
L-Phe:L-Lys	768.2345	–2619.1
L-Glu:L-Asp:L-Pro	617.3223	1345.4
L-Phe	491.5065	471.338
L-Glu:L-Phe	665.2995	–1084.7

## 4 Discussion

The findings of this paper shed light on the potential functions of proteinoids in neuronal circuitry, ranging from providing structure and format for electrical signals to acting as mediators in the transmission of physiological information. It is now possible to investigate communication in biological compounds through electrical oscillations and compare the results with those observed in more complex biological systems. The discussion section will delve deeper into the potential impact of this work on understanding the function of proteinoids in neuronal signaling and its implications for ongoing research into electrical communication in living organisms.

Recent research suggests a correlation between proteinoid oscillations and communication, similar to the correlation discovered by Adamatzky et al.^36^ in their investigation of oscillations in fungi. Communication between microspheres is crucial for the development and evolution of complex systems like unconventional computing and autonomous robotics. The nervous system of proteinoid microspheres and their analogues provide insights into their interactions.

Communication between microspheres primarily occurs through direct contact, allowing signal transmission through excitability. This involves the spheres coming into contact through surface tension or mechanical pressure (piezoelectricity). While this approach is reliable, chemical differences among the microspheres may hinder it. Electrical coupling is the most common method of communication between microspheres. It enables the transmission of binary information, such as digitally encoded data packets, using electrical signals. Proteinoid microspheres have the ability to communicate and share information using different methods such as electromagnetic, optical, and chemical signaling. Electromagnetic coupling is based on the generation of inductive currents between microspheres. A single vesicle functions as the transmitter by producing a modulated electromagnetic field that then generates a current in the receiver microsphere. Wireless transmission enables the encoding and transmission of information through electromagnetic waves. Hybrid proteinoid inorganic compositions with tunable photoresponsive properties are used for optical coupling. These compositions allow for the modulation of absorption and scattering of light signals. The optical pulses are transduced into detectable signals in the receiving vesicle through photoreactions in the proteinoids or plasmonic responses in inorganic nanoparticles. This technology enables the exchange of high-bandwidth wireless data using optical methods. Chemical signaling involves the release and diffusion of molecular messengers such as protons, ions, or organic molecules. The microsphere that receives the signals has receptors that can detect and respond to the chemical signals emitted by the microsphere that transmits them. The localized proteinoid–proteinoid interactions are made possible by this molecular communication channel.^37^ Communication among microspheres can be categorized into two distinct categories: information exchange and control. Information exchange involves the transmission and reception of data, while control refers to the transmission and reception of commands. Microspheres engage in information exchange, interaction, and resource sharing to facilitate the advancement of complex systems and procedures.

**Figure 8:**
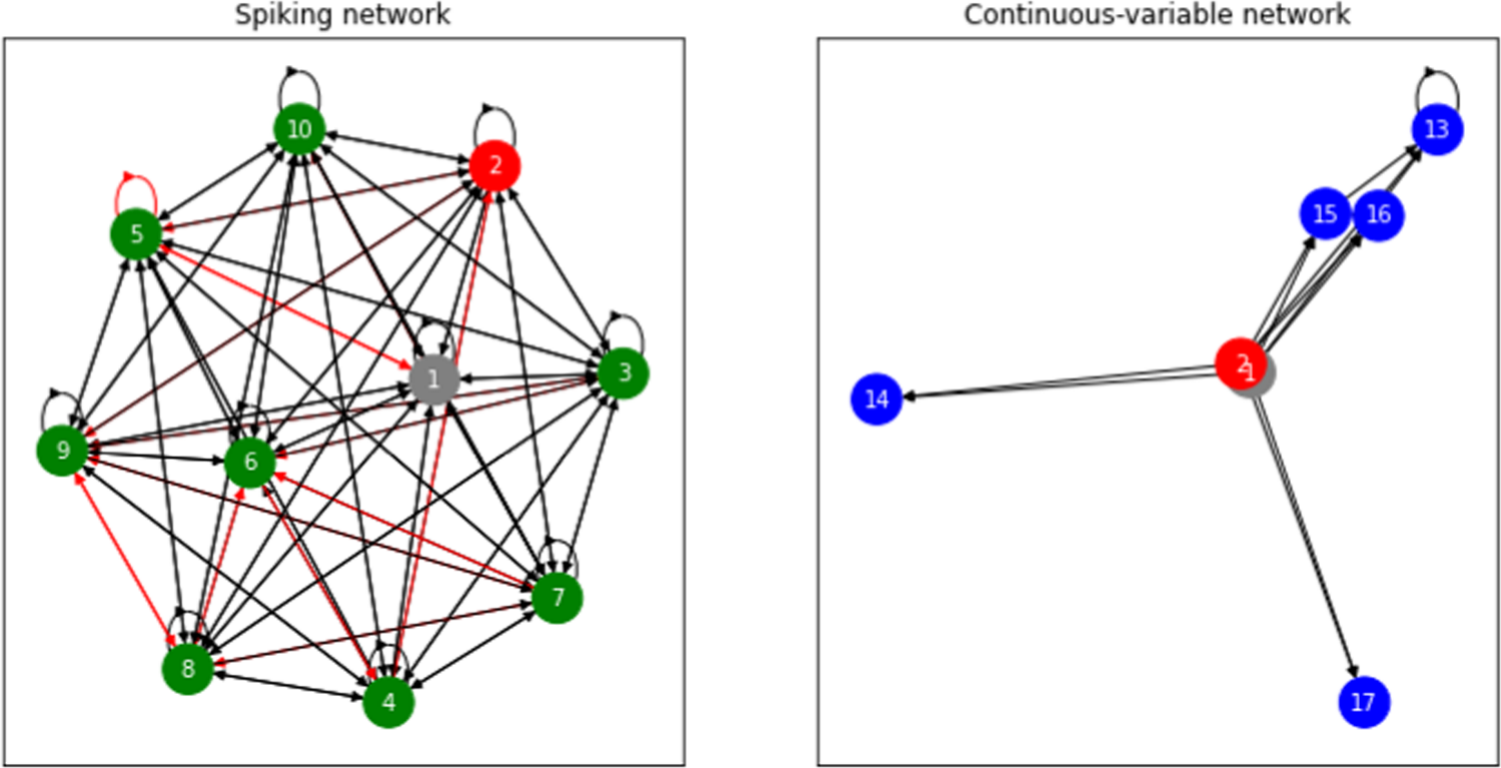
Network architectures of proteinoid microspheres. (a) Spiking network. A network of N recurrently connected leaky integrate-and-fire neurons (green circles) receives an input *F*_in_(*t*) (gray circle) through synapse U and generates an output *F*_out_(*t*) (red circle) through synapse W. (b) Continuous-variable network. A network of *N*_tilde_ recurrently connected “rate” units (blue circles) receive inputs *F*_in_(*t*) and *F*_out_(*t*) through synapses *U*_tilde_ and *u*, respectively.^38^

Figure 8 provides insights into potential interpretations and analogies of a proteinoid microsphere nervous system. It offers an understanding of microspheres’ interactions and network organization similar to biological nervous systems. The figure presents two distinct architectures showcasing the potential functions of proteinoid microspheres. The first architecture depicts spiking networks composed of leaky integrate-and-fire neurons that receive external input force *F*_in_(*t*) and produce output *F*_out_(*t*) via synapses W. This architecture resembles the nervous system of advanced organisms, as the input and output signals exhibit similar behavior and generation patterns to those found in a typical nervous system. The second architecture utilizes continuous-variable networks to process an input from an external force *F*_in_(*t*) and an internal output *F*_out_(*t*) to produce the corresponding output. Continuous variables are employed instead of binary states of neurons, allowing for a wider range of interpretations and analogies of the nervous system. This network architecture provides a more realistic representation of the nervous system and its associated functions.

Proteinoid microspheres have the potential to function as protoneural networks, as shown in Figure 8, with their two distinct architectures. Proteinoid microspheres serve as the fundamental units of the network, enabling basic communication and potential capacity for simple computations. As the network expands, it can develop intricate architectures that leverage the inherent connectivity of interconnected molecules, enabling significantly advanced functions and capabilities. This distinguishes proteinoid microsphere networks from conventional computing architectures that rely on external wiring for communication.

Proteinoid microspheres and biological neurons have distinct compositions, architectures, and functional mechanisms. However, they do share certain broad similarities in their emergent properties. Proteinoid vesicles can display spontaneous oscillations and propagating excitation waves reminiscent of neural action potentials. However, these lack the complex voltage-gated ion channel dynamics of biological neurons. Networks of proteinoid microspheres show collective synchronization behaviors analogous to clustering and neural network-level signaling. However, biological synaptic connectivity involves intricate molecular machinery not present in the proteinoid systems. Proteinoid microspheres are artificially synthesized structures made up of chemically bonded amino acids created in a laboratory setting. In contrast, neural networks are complex biological systems composed of individual neurons that work together in a coordinated manner. Proteinoid microspheres exhibit a less complex architecture compared to neural networks, with each node accountable for a single function, while neurons can process multiple inputs and perform various roles, such as transmitting signals between neurons or serving as synapses. Proteinoid microspheres have limited behavioral capabilities, primarily focused on simple tasks like self-repair and shape adaptation due to their inherent lack of complexity. In contrast, biological neural networks possess advanced capabilities such as memory formation, decision-making, and learning. While both proteinoid microspheres and biological neural networks can process information, the intricate and adaptable nature of biological neural networks sets them apart from proteinoid microspheres.^39−42^

The results obtained from detecting spikes in proteinoid microspheres using differential pulse voltammetry (DPV) demonstrate electrical excitability and signal transmission capabilities. While intriguing, significantly more investigation is required to determine any potential relevance of these properties to the self-organizing systems thought to be precursors to the origins of life. Proteinoid microspheres possess the property of self-assembly, allowing for the aggregation of essential elements necessary for the genesis of protocells and the formation of intricate structures. Additionally, the microspheres have the ability to retain and convey data, a crucial prerequisite for the origin of biological existence. The collective capabilities of proteinoid microspheres may have facilitated the emergence and development of primitive cells during the initial phases of life.

Proteinoid microspheres offer a fresh perspective for advancing our understanding of neural circuits. As researchers delve deeper into the system’s adaptability, it is anticipated that proteinoids will unlock new insights in currently unexplored domains. These discoveries have the potential to pave the way for improved treatments for neurological disorders and advancements in medical technology and unconventional computing.

## 5 Conclusions

The findings of the study highlight the promising compatibility between differential pulse voltammetry and proteinoid nanobrains, opening up a new avenue for exploring these unique systems. The results suggest that utilizing differential pulse voltammetry as a tool can greatly contribute to understanding the functionality of proteinoid nanobrains, offering valuable insights into their behavior and potential applications. Further research in this area could unlock a deeper comprehension of these nanobrains and their potential role in the development of intelligent machines, potentially revolutionizing the field of artificial intelligence.
